# [(Pyrrolidin-1-yl)carbothio­ylsulfan­yl]methyl pyrrolidine-1-carbodithio­ate

**DOI:** 10.1107/S1600536810046027

**Published:** 2010-11-17

**Authors:** Wei-Lung Chou, Kuang-Hway Yih, Gene-Hsiang Lee, Yen-Hsiang Huang, Hsiao-Fen Wang

**Affiliations:** a Department of Industrial Safety and Health & Institute Occupational Safety and Hazard Prevention, Hungkuang University, Shalu 433, Taichung, Taiwan; b Department of Applied Cosmetology, Hungkuang University, Shalu 433, Taichung, Taiwan; c Instrumentation Center, College of Science, National Taiwan University, Taipei 106, Taiwan

## Abstract

The title compound, C_11_H_18_N_2_S_4_, was unexpectedly obtained during studies on the reactivity of the complex tris­(acac-κ^2^ *O*,*O*′)gallium(III) (acac is acetyl­acetonate) with C_4_H_8_NCS_2_H in dichloro­methane. The title compound shows disordered two pyrrolidine rings with major and minor occupancies of 0.546 (4) and 0.454 (4). Two (pyrrolidin-1-yl)carbothio­ylsulfanyl units are linked together through a methyl­ene C atom and weak C—H⋯S inter­actions are found.

## Related literature

For bis­(dialkyl­dithio­carbamates), CH_2_(S_2_CN*R*_2_)_2_, see: *R* = Me (Thomas, 1945 ▶, 1946 ▶); *R* = Et (Heckley *et al.*, 1970 ▶); *R* = C_5_H_10_ (Sharma *et al.*, 1991 ▶). For weak C—H⋯S inter­actions, see: Kayed *et al.* (2008 ▶); Pervez *et al.* (2010 ▶); Vangala *et al.* (2002 ▶); Yaqub *et al.* (2010 ▶). For our previous work on the preparation of In(III) complexes, see: Chou *et al.* (2007 ▶). For C=S double-bond lengths, see: Pauling (1960 ▶).
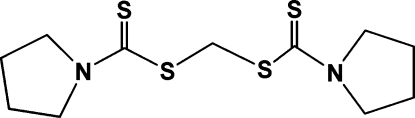


## Experimental

### 

#### Crystal data

C_11_H_18_N_2_S_4_
*M* *_r_* = 306.51
Orthorhombic, 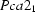

*a* = 21.9118 (18) Å
*b* = 4.5705 (4) Å
*c* = 14.3452 (12) Å
*V* = 1436.6 (2) Å^3^
*Z* = 4
Mo *K*α radiation
μ = 0.64 mm^−1^
*T* = 150 K
0.25 × 0.25 × 0.15 mm

#### Data collection

Bruker SMART APEX CCD area-detector diffractometer
Absorption correction: multi-scan (*SADABS*; Bruker, 2001 ▶) *T*_min_ = 0.856, *T*_max_ = 0.910
17016 measured reflections
3292 independent reflections
2759 reflections with *I* > 2σ(*I*)
*R*_int_ = 0.043

#### Refinement

*R*[*F* ^2^ > 2σ(*F* ^2^)] = 0.062
*wR*(*F* ^2^) = 0.166
*S* = 1.07
3292 reflections
180 parameters
17 restraints
H-atom parameters constrained
Δρ_max_ = 1.05 e Å^−3^
Δρ_min_ = −0.27 e Å^−3^
Absolute structure: Flack (1983 ▶), 1579 Friedel pairs
Flack parameter: −0.1 (2)

### 

Data collection: *SMART* (Bruker, 2007 ▶); cell refinement: *SAINT* (Bruker, 2007 ▶); data reduction: *SAINT*; program(s) used to solve structure: *SHELXS97* (Sheldrick, 2008 ▶); program(s) used to refine structure: *SHELXL97* (Sheldrick, 2008 ▶); molecular graphics: *XP* in *SHELXTL* (Sheldrick, 2008 ▶); software used to prepare material for publication: *SHELXTL*.

## Supplementary Material

Crystal structure: contains datablocks I, global. DOI: 10.1107/S1600536810046027/bv2162sup1.cif

Structure factors: contains datablocks I. DOI: 10.1107/S1600536810046027/bv2162Isup2.hkl

Additional supplementary materials:  crystallographic information; 3D view; checkCIF report

## Figures and Tables

**Table 1:** Hydrogen-bond geometry (Å, °)

*D*—H⋯*A*	*D*—H	H⋯*A*	*D*⋯*A*	*D*—H⋯*A*
C4—H4*A*⋯S3^i^	0.99	2.89	3.811 (9)	155
C10—H10*B*⋯S4^ii^	0.99	2.99	3.699 (11)	130
C9—H9*A*⋯S1^ii^	0.99	2.87	3.740 (8)	147
C9′—H9′*A*⋯S1^ii^	0.99	3.50	4.209 (11)	131
C5′—H5′*B*⋯S2^i^	0.99	2.94	3.704 (14)	137

Symmetry codes: (i) 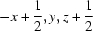; (ii) 
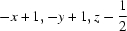
.

**Fig. 1.**
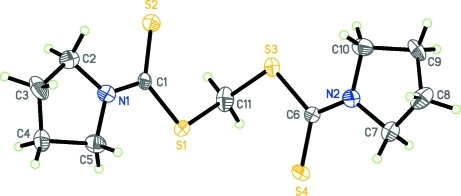
The molecular structure of (I), showing two independent molecules and the 50% probability displacement ellipsoids.

**Fig. 2.**
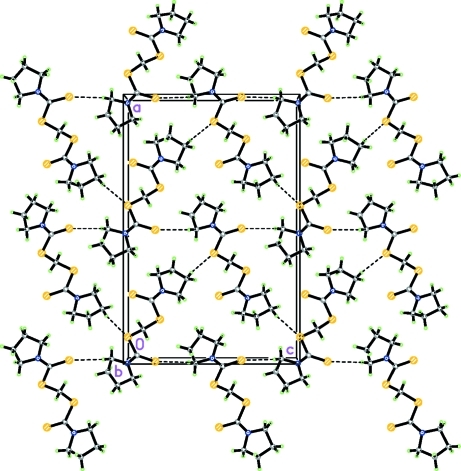
The packing diagram of (I), showing two different packing patterns.

## supplementary crystallographic information

### Comment

Formation of methylene bis(dialkyldithiocarbamates), CH_2_(S_2_CN*R*_2_)_2_ [*R* = Me (Thomas, 1946), Et (Heckley *et al.*, 1970), C_5_H_10_ (Sharma *et al.*, 1991)] have been reported in the literature as by-products in the reactions of transition metal halides with anhydrous sodium dialkyldithiocarbamates when methylene chloride was used as solvent or reaction of anhydrous sodium dialkyldithiocarbamates with methylene chloride under refluxing conditions (Sharma *et al.*, 1991).

Our previous report showed complexes [In(S_2_CNC_5_H_10_)_3_], [In(pyS)_3_] and [In(pyS)_2_(acac)] (acac: acetylacetonate; pyS: pyridine-2-thionate) are prepared by reacting the complex tris(acac-κ^2^*O*,*O*')indium(III) with HS_2_CNC_5_H_10_, and pySH with ratios of 1:3, 1:3, and 1:2 in dichloromethane at room temperature, respectively (Chou *et al.*, 2007). To test the generality of this substitution reaction, we studied the reaction of tris(acac-κ^2^*O*,*O*')gallium(III) complex and C_4_H_8_NCS_2_H. During studies on the reactivity of complex tris(acac-κ^2^*O*,*O*')gallium(III), with C_4_H_8_NCS_2_H in dichloromethane, we unexpectedly obtained the white crystals of title compound (I), identified as methylene bis(pyrrolidinyldithiocarbamate) by X-ray structure, NMR and Mass spectroscopic analyses. It consists of two pyrrolidinyldithiocarbamate units, bridged by a methylene group, *i.e.* C_4_H_8_N—CS—S—CH_2_—S—CS—NC_4_H_8_. The ^1^H NMR spectrum of (I) in CDCl_3_ shows one singlet at 5.33 ppm., assignable to SCH_2_S. The IR spectrum shows the following characteristic bands, 1470 cm^-1^ (νC=N), 1305 cm^-1^ (νC-N), 990 cm^-1^ (νC=S), 915 cm^-1^ (νC-S). The FAB mass spectrum shows the molecular ions C_11_H_18_N_2_S_4_ with the characteristic isotopic distribution patterns.

The solid-state structure has been established by X-ray crystallography. The molecular structure of the title compound is shown in Fig. 1. In (I), the C1—S2 and C6—S4 bond lengths of 1.725 (10) and 1.693 (8) Å, respectively, are slightly longer than a normal C=S double bond (*ca* 1.61 Å) (Pauling, 1960), while the C1—S1 and C6—S3 distance of 1.743 (10) and 1.827 (8) Å, respectively, are clearly single bonds. The angle of S3—C11—S1 (114.05 (18)°) is larger than the ideal tetrahedral value of 109.47°, probably due to repulsion between the two C=S bonding electron pairs. Two pyrrolidinyl groups are found to be disordered over two positions (C1, C2, C3, C4, C5, C6, C7, C8, C9, C10) and (C1', C2', C3', C4', C5', C6', C7', C8', C9', C10') and refined ratios of the major and minor components being 0.546 (4): 0.454 (4). As a result of two different packings are shown in Fig. 2(*a*) and (*b*). The weak interactions of C—H···S (3.683 (6) - 3.823 (11) Å) in (I) are also found in those of (*E*)-2-[1-(1-benzothiophen-3-yl)ethylidene]hydrazinecarbothioamide (3.613 (3) - 3.762 (4) Å) (Kayed *et al.*, 2008), 4-(5-chloro-2- methylphenyl)-1-[2-oxo-5-(trifluoromethoxy)indolin-3-ylidene]thiosemicarbazide (3.245 (4) Å) (Pervez *et al.*, 2010), bis(4-aminophenyl)disulfide (3.7387 (18) Å) (Vangala *et al.*, 2002) and 1-[1-(4-bromophenyl)ethylidene]-4-(2,4-dimethoxyphenyl)thiosemicarbazide (3.774 (3) Å) (Yaqub *et al.*, 2010), respectively.

### Experimental

The synthesis of the title compound (I) was carried out as follows. 10 ml of CH_2_Cl_2_ was added to a flask of Ga(acac)_3_ (0.367 g, 1.0 mmol) and C_4_H_8_NCS_2_H (0.345 g, 3.0 mmol). The solution was stirred for 2 days at room temperature. The solution is concentrated under vacuum and n-hexane (10 ml) was added to initiate precipitation. The pale-white solids were isolated by filtration (G4), washed with n-hexane (2 *x* 10 ml) and subsequently drying under vacuum yielding [CH_2_(S_2_CNC_4_H_8_)_2_] (0.459 g, 50%). Further purification was accomplished by recrystallization from 1/10 CH_2_Cl_2_/n-hexane. The pale-white crystals of (I) for X-ray structure analysis were obtained by slow diffusion of n-hexane into the CH_2_Cl_2_ solution of the title compound at room temperature for 3 days. Spectroscopic analysis: ^1^H NMR (CDCl_3_, 298 K, δ, p.p.m.): δ 1.65, 1.74 (m, 4H, NCCH_2_), δ 2.98, 3.29 (m, 4H, NCH_2_), 5.33 (s, 2H, SCH_2_). 13C{^1^H} NMR (CDCl_3_, 298 K, δ, p.p.m.): δ 24.8 (s, NCH_2_CH_2_), 49.8 (s, NCH_2_), 50.0 (s, SCH_2_S), 191.5 (s, CS). MS (m/*z*): 306.5 (*M*^+^). Anal. Calcd for C_11_H_18_N_2_S_4_: C, 43.10; H, 5.92; N, 9.14. Found: C, 43.31; H, 5.69; N, 9.02.

### Refinement

Two pyrrolidinyl groups are found to be disordered over two positions (C1, C2, C3, C4, C5, C6, C7, C8, C9, C10) and (C1', C2', C3', C4', C5', C6', C7', C8', C9', C10') and the occupancies are refined to 0.546 (4) and 0.454 (4).

H atoms were positioned geometrically and refined using a riding model, with C—H = 0.99 Å and with *U*_iso_(H) = 1.2 times *U*_eq_(C).

### Crystal data

**Table:**

C_11_H_18_N_2_S_4_	*F*(000) = 648
*M**_r_* = 306.51	*D*_x_ = 1.417 Mg m^−^^3^
Orthorhombic, *P**c**a*2_1_	Mo *K*α radiation, λ = 0.71073 Å
Hall symbol: P 2c -2ac	Cell parameters from 2540 reflections
*a* = 21.9118 (18) Å	θ = 2.3–26.6°
*b* = 4.5705 (4) Å	µ = 0.64 mm^−^^1^
*c* = 14.3452 (12) Å	*T* = 150 K
*V* = 1436.6 (2) Å^3^	Block, light-brown
*Z* = 4	0.25 × 0.25 × 0.15 mm

### Data collection

**Table:**

Bruker SMART APEX CCD area-detector diffractometer	3292 independent reflections
Radiation source: fine-focus sealed tube	2759 reflections with *I* > 2σ(*I*)
graphite	*R*_int_ = 0.043
ω scans	θ_max_ = 27.5°, θ_min_ = 1.9°
Absorption correction: multi-scan (*SADABS*; Bruker, 2001)	*h* = −28→28
*T*_min_ = 0.856, *T*_max_ = 0.910	*k* = −5→5
17016 measured reflections	*l* = −18→18

### Refinement

**Table:**

Refinement on *F*^2^	Secondary atom site location: difference Fourier map
Least-squares matrix: full	Hydrogen site location: inferred from neighbouring sites
*R*[*F*^2^ > 2σ(*F*^2^)] = 0.062	H-atom parameters constrained
*wR*(*F*^2^) = 0.166	*w* = 1/[σ^2^(*F*_o_^2^) + (0.1007*P*)^2^ + 0.6346*P*] where *P* = (*F*_o_^2^ + 2*F*_c_^2^)/3
*S* = 1.07	(Δ/σ)_max_ = 0.003
3292 reflections	Δρ_max_ = 1.05 e Å^−^^3^
180 parameters	Δρ_min_ = −0.27 e Å^−^^3^
17 restraints	Absolute structure: Flack (1983), 1579 Friedel pairs
Primary atom site location: structure-invariant direct methods	Flack parameter: −0.1 (2)

### Special details

**Table:**

Geometry. All e.s.d.'s (except the e.s.d. in the dihedral angle between two l.s. planes) are estimated using the full covariance matrix. The cell e.s.d.'s are taken into account individually in the estimation of e.s.d.'s in distances, angles and torsion angles; correlations between e.s.d.'s in cell parameters are only used when they are defined by crystal symmetry. An approximate (isotropic) treatment of cell e.s.d.'s is used for estimating e.s.d.'s involving l.s. planes.
Refinement. Refinement of *F*^2^ against ALL reflections. The weighted *R*-factor *wR* and goodness of fit *S* are based on *F*^2^, conventional *R*-factors *R* are based on *F*, with *F* set to zero for negative *F*^2^. The threshold expression of *F*^2^ > σ(*F*^2^) is used only for calculating *R*-factors(gt) *etc*. and is not relevant to the choice of reflections for refinement. *R*-factors based on *F*^2^ are statistically about twice as large as those based on *F*, and *R*- factors based on ALL data will be even larger.

### Fractional atomic coordinates and isotropic or equivalent isotropic displacement parameters (Å^2^)

**Table:**

	*x*	*y*	*z*	*U*_iso_*/*U*_eq_	Occ. (<1)
S1	0.34241 (4)	0.48896 (18)	0.20742 (6)	0.0247 (3)	
S2	0.24742 (5)	0.4861 (2)	0.05150 (8)	0.0332 (3)	
S3	0.40977 (4)	0.48200 (19)	0.02225 (7)	0.0292 (3)	
S4	0.50250 (6)	0.4996 (2)	0.18040 (8)	0.0333 (3)	
N1	0.23596 (13)	0.7324 (7)	0.2179 (2)	0.0280 (7)	
N2	0.51532 (12)	0.7401 (7)	0.0131 (2)	0.0292 (7)	
C1	0.2724 (4)	0.593 (2)	0.1603 (7)	0.0232 (12)	0.546 (4)
C2	0.1742 (5)	0.840 (3)	0.1934 (7)	0.0354 (9)	0.546 (4)
H2A	0.1450	0.6753	0.1894	0.043*	0.546 (4)
H2B	0.1749	0.9430	0.1327	0.043*	0.546 (4)
C3	0.1558 (4)	1.0519 (19)	0.2724 (6)	0.0388 (19)	0.546 (4)
H3A	0.1691	1.2541	0.2580	0.047*	0.546 (4)
H3B	0.1111	1.0507	0.2820	0.047*	0.546 (4)
C4	0.1889 (4)	0.935 (2)	0.3579 (6)	0.039 (2)	0.546 (4)
H4A	0.1669	0.7670	0.3857	0.047*	0.546 (4)
H4B	0.1943	1.0887	0.4057	0.047*	0.546 (4)
C5	0.2514 (4)	0.838 (2)	0.3155 (7)	0.0288 (11)	0.546 (4)
H5A	0.2803	1.0043	0.3132	0.035*	0.546 (4)
H5B	0.2698	0.6784	0.3528	0.035*	0.546 (4)
C1'	0.2682 (6)	0.549 (3)	0.1543 (10)	0.0232 (12)	0.454 (4)
C2'	0.1763 (6)	0.843 (4)	0.1926 (8)	0.0354 (9)	0.454 (4)
H2'A	0.1520	0.6948	0.1589	0.043*	0.454 (4)
H2'B	0.1794	1.0226	0.1541	0.043*	0.454 (4)
C3'	0.1491 (4)	0.910 (2)	0.2897 (7)	0.0388 (19)	0.454 (4)
H3'A	0.1174	1.0640	0.2862	0.047*	0.454 (4)
H3'B	0.1314	0.7321	0.3184	0.047*	0.454 (4)
C4'	0.2053 (4)	1.016 (2)	0.3437 (9)	0.039 (2)	0.454 (4)
H4'A	0.1985	1.0058	0.4119	0.047*	0.454 (4)
H4'B	0.2164	1.2183	0.3262	0.047*	0.454 (4)
C5'	0.2549 (5)	0.793 (3)	0.3119 (10)	0.0288 (11)	0.454 (4)
H5'A	0.2962	0.8811	0.3136	0.035*	0.454 (4)
H5'B	0.2544	0.6145	0.3508	0.035*	0.454 (4)
C6	0.4816 (3)	0.6102 (15)	0.0724 (6)	0.0200 (9)*	0.546 (4)
C7	0.5772 (5)	0.855 (3)	0.0389 (7)	0.0366 (10)	0.546 (4)
H7A	0.6075	0.6954	0.0428	0.044*	0.546 (4)
H7B	0.5760	0.9611	0.0990	0.044*	0.546 (4)
C8	0.5912 (4)	1.0600 (16)	−0.0409 (6)	0.033 (2)	0.546 (4)
H8A	0.5747	1.2579	−0.0287	0.040*	0.546 (4)
H8B	0.6358	1.0747	−0.0513	0.040*	0.546 (4)
C9	0.5603 (4)	0.9224 (18)	−0.1231 (5)	0.0301 (17)	0.546 (4)
H9A	0.5837	0.7523	−0.1464	0.036*	0.546 (4)
H9B	0.5551	1.0652	−0.1743	0.036*	0.546 (4)
C10	0.4984 (4)	0.827 (3)	−0.0837 (8)	0.0322 (10)	0.546 (4)
H10A	0.4687	0.9902	−0.0839	0.039*	0.546 (4)
H10B	0.4813	0.6600	−0.1191	0.039*	0.546 (4)
C6'	0.4807 (5)	0.551 (2)	0.0695 (8)	0.0200 (9)*	0.454 (4)
C7'	0.5740 (6)	0.853 (4)	0.0417 (8)	0.0366 (10)	0.454 (4)
H7'A	0.5983	0.7013	0.0740	0.044*	0.454 (4)
H7'B	0.5693	1.0245	0.0832	0.044*	0.454 (4)
C8'	0.6031 (4)	0.939 (2)	−0.0505 (8)	0.033 (2)	0.454 (4)
H8'A	0.6318	1.1039	−0.0417	0.040*	0.454 (4)
H8'B	0.6256	0.7717	−0.0778	0.040*	0.454 (4)
C9'	0.5511 (5)	1.027 (2)	−0.1120 (8)	0.0301 (17)	0.454 (4)
H9'A	0.5618	1.0074	−0.1788	0.036*	0.454 (4)
H9'B	0.5383	1.2315	−0.0996	0.036*	0.454 (4)
C10'	0.5010 (5)	0.808 (4)	−0.0837 (10)	0.0322 (10)	0.454 (4)
H10C	0.4599	0.8968	−0.0892	0.039*	0.454 (4)
H10D	0.5027	0.6299	−0.1230	0.039*	0.454 (4)
C11	0.37625 (18)	0.2698 (7)	0.1150 (4)	0.0358 (8)	
H11A	0.4081	0.1426	0.1423	0.043*	
H11B	0.3444	0.1413	0.0882	0.043*	

### Atomic displacement parameters (Å^2^)

**Table:**

	*U*^11^	*U*^22^	*U*^33^	*U*^12^	*U*^13^	*U*^23^
S1	0.0171 (4)	0.0341 (5)	0.0230 (6)	0.0012 (3)	−0.0019 (4)	0.0022 (3)
S2	0.0255 (5)	0.0514 (7)	0.0228 (6)	−0.0023 (4)	0.0000 (4)	−0.0059 (4)
S3	0.0214 (5)	0.0404 (6)	0.0259 (7)	−0.0009 (3)	0.0048 (4)	−0.0009 (4)
S4	0.0291 (5)	0.0535 (8)	0.0174 (6)	0.0030 (4)	0.0006 (4)	0.0072 (4)
N1	0.0272 (14)	0.0342 (15)	0.0226 (15)	0.0002 (12)	0.0001 (12)	0.0005 (12)
N2	0.0245 (13)	0.0388 (17)	0.0244 (15)	0.0028 (12)	0.0007 (12)	0.0005 (13)
C1	0.0175 (18)	0.031 (3)	0.0217 (19)	−0.0108 (19)	0.0036 (15)	0.003 (2)
C2	0.0293 (18)	0.049 (2)	0.0284 (18)	0.0045 (17)	−0.0025 (15)	−0.0022 (18)
C3	0.044 (3)	0.042 (5)	0.030 (4)	0.011 (3)	0.016 (3)	0.014 (3)
C4	0.026 (4)	0.059 (5)	0.033 (4)	−0.008 (3)	0.008 (3)	−0.002 (3)
C5	0.0359 (19)	0.025 (3)	0.0251 (18)	0.0014 (19)	−0.0016 (16)	−0.007 (2)
C1'	0.0175 (18)	0.031 (3)	0.0217 (19)	−0.0108 (19)	0.0036 (15)	0.003 (2)
C2'	0.0293 (18)	0.049 (2)	0.0284 (18)	0.0045 (17)	−0.0025 (15)	−0.0022 (18)
C3'	0.044 (3)	0.042 (5)	0.030 (4)	0.011 (3)	0.016 (3)	0.014 (3)
C4'	0.026 (4)	0.059 (5)	0.033 (4)	−0.008 (3)	0.008 (3)	−0.002 (3)
C5'	0.0359 (19)	0.025 (3)	0.0251 (18)	0.0014 (19)	−0.0016 (16)	−0.007 (2)
C7	0.0239 (18)	0.053 (3)	0.033 (2)	−0.0054 (17)	−0.0006 (16)	0.0003 (19)
C8	0.028 (3)	0.020 (5)	0.052 (5)	0.003 (3)	−0.010 (3)	0.009 (3)
C9	0.039 (3)	0.030 (5)	0.021 (3)	0.005 (3)	0.013 (3)	0.000 (3)
C10	0.037 (2)	0.036 (2)	0.0245 (19)	0.0014 (17)	−0.0037 (15)	−0.0016 (17)
C7'	0.0239 (18)	0.053 (3)	0.033 (2)	−0.0054 (17)	−0.0006 (16)	0.0003 (19)
C8'	0.028 (3)	0.020 (5)	0.052 (5)	0.003 (3)	−0.010 (3)	0.009 (3)
C9'	0.039 (3)	0.030 (5)	0.021 (3)	0.005 (3)	0.013 (3)	0.000 (3)
C10'	0.037 (2)	0.036 (2)	0.0245 (19)	0.0014 (17)	−0.0037 (15)	−0.0016 (17)
C11	0.0294 (15)	0.0314 (17)	0.047 (2)	−0.0009 (17)	0.0098 (14)	0.003 (2)

### Geometric parameters (Å, °)

**Table:**

S1—C1	1.743 (10)	C2'—H2'A	0.9900
S1—C1'	1.816 (14)	C2'—H2'B	0.9900
S1—C11	1.820 (5)	C3'—C4'	1.533 (12)
S2—C1'	1.571 (14)	C3'—H3'A	0.9900
S2—C1	1.725 (10)	C3'—H3'B	0.9900
S3—C6'	1.724 (11)	C4'—C5'	1.556 (12)
S3—C11	1.802 (5)	C4'—H4'A	0.9900
S3—C6	1.827 (8)	C4'—H4'B	0.9900
S4—C6'	1.678 (12)	C5'—H5'A	0.9900
S4—C6	1.693 (8)	C5'—H5'B	0.9900
N1—C1	1.313 (10)	C7—C8	1.511 (11)
N1—C1'	1.426 (13)	C7—H7A	0.9900
N1—C5'	1.438 (13)	C7—H7B	0.9900
N1—C2'	1.448 (14)	C8—C9	1.498 (10)
N1—C2	1.483 (11)	C8—H8A	0.9900
N1—C5	1.520 (10)	C8—H8B	0.9900
N2—C6	1.273 (8)	C9—C10	1.531 (10)
N2—C6'	1.406 (11)	C9—H9A	0.9900
N2—C7'	1.445 (13)	C9—H9B	0.9900
N2—C10'	1.457 (14)	C10—H10A	0.9900
N2—C10	1.491 (11)	C10—H10B	0.9900
N2—C7	1.500 (11)	C7'—C8'	1.520 (12)
C2—C3	1.543 (11)	C7'—H7'A	0.9900
C2—H2A	0.9900	C7'—H7'B	0.9900
C2—H2B	0.9900	C8'—C9'	1.498 (11)
C3—C4	1.522 (10)	C8'—H8'A	0.9900
C3—H3A	0.9900	C8'—H8'B	0.9900
C3—H3B	0.9900	C9'—C10'	1.541 (12)
C4—C5	1.563 (10)	C9'—H9'A	0.9900
C4—H4A	0.9900	C9'—H9'B	0.9900
C4—H4B	0.9900	C10'—H10C	0.9900
C5—H5A	0.9900	C10'—H10D	0.9900
C5—H5B	0.9900	C11—H11A	0.9900
C2'—C3'	1.546 (12)	C11—H11B	0.9900
			
C1—S1—C1'	7.3 (7)	C2'—C3'—H3'A	111.4
C1—S1—C11	103.1 (4)	C4'—C3'—H3'B	111.4
C1'—S1—C11	98.2 (5)	C2'—C3'—H3'B	111.4
C1'—S2—C1	6.3 (9)	H3'A—C3'—H3'B	109.2
C6'—S3—C11	100.1 (4)	C3'—C4'—C5'	101.9 (9)
C6'—S3—C6	8.2 (4)	C3'—C4'—H4'A	111.4
C11—S3—C6	103.5 (3)	C5'—C4'—H4'A	111.4
C6'—S4—C6	9.3 (5)	C3'—C4'—H4'B	111.4
C1—N1—C1'	8.8 (10)	C5'—C4'—H4'B	111.4
C1—N1—C5'	120.5 (7)	H4'A—C4'—H4'B	109.2
C1'—N1—C5'	124.8 (7)	N1—C5'—C4'	101.6 (8)
C1—N1—C2'	124.2 (7)	N1—C5'—H5'A	111.5
C1'—N1—C2'	119.6 (7)	C4'—C5'—H5'A	111.5
C5'—N1—C2'	115.3 (6)	N1—C5'—H5'B	111.5
C1—N1—C2	124.6 (6)	C4'—C5'—H5'B	111.5
C1'—N1—C2	119.8 (7)	H5'A—C5'—H5'B	109.3
C5'—N1—C2	114.9 (6)	N2—C6—S4	126.4 (5)
C2'—N1—C2	1.5 (13)	N2—C6—S3	112.7 (5)
C1—N1—C5	126.7 (6)	S4—C6—S3	119.8 (4)
C1'—N1—C5	131.7 (7)	N2—C7—C8	102.4 (7)
C5'—N1—C5	8.1 (8)	N2—C7—H7A	111.3
C2'—N1—C5	108.7 (6)	C8—C7—H7A	111.3
C2—N1—C5	108.4 (5)	N2—C7—H7B	111.3
C6—N2—C6'	10.3 (7)	C8—C7—H7B	111.3
C6—N2—C7'	119.5 (6)	H7A—C7—H7B	109.2
C6'—N2—C7'	122.4 (7)	C9—C8—C7	104.1 (7)
C6—N2—C10'	127.7 (6)	C9—C8—H8A	110.9
C6'—N2—C10'	124.3 (7)	C7—C8—H8A	110.9
C7'—N2—C10'	112.7 (6)	C9—C8—H8B	110.9
C6—N2—C10	127.1 (5)	C7—C8—H8B	110.9
C6'—N2—C10	124.5 (6)	H8A—C8—H8B	109.0
C7'—N2—C10	113.0 (6)	C8—C9—C10	103.3 (7)
C10'—N2—C10	3.8 (12)	C8—C9—H9A	111.1
C6—N2—C7	121.5 (5)	C10—C9—H9A	111.1
C6'—N2—C7	124.1 (6)	C8—C9—H9B	111.1
C7'—N2—C7	2.3 (9)	C10—C9—H9B	111.1
C10'—N2—C7	110.8 (6)	H9A—C9—H9B	109.1
C10—N2—C7	111.1 (5)	N2—C10—C9	101.5 (6)
N1—C1—S2	120.9 (7)	N2—C10—H10A	111.5
N1—C1—S1	115.1 (7)	C9—C10—H10A	111.5
S2—C1—S1	123.6 (6)	N2—C10—H10B	111.5
N1—C2—C3	105.8 (7)	C9—C10—H10B	111.5
N1—C2—H2A	110.6	H10A—C10—H10B	109.3
C3—C2—H2A	110.6	N2—C6'—S4	118.6 (7)
N1—C2—H2B	110.6	N2—C6'—S3	111.9 (7)
C3—C2—H2B	110.6	S4—C6'—S3	127.1 (6)
H2A—C2—H2B	108.7	N2—C7'—C8'	102.7 (8)
C4—C3—C2	104.3 (7)	N2—C7'—H7'A	111.2
C4—C3—H3A	110.9	C8'—C7'—H7'A	111.2
C2—C3—H3A	110.9	N2—C7'—H7'B	111.2
C4—C3—H3B	110.9	C8'—C7'—H7'B	111.2
C2—C3—H3B	110.9	H7'A—C7'—H7'B	109.1
H3A—C3—H3B	108.9	C9'—C8'—C7'	105.2 (9)
C3—C4—C5	101.8 (7)	C9'—C8'—H8'A	110.7
C3—C4—H4A	111.4	C7'—C8'—H8'A	110.7
C5—C4—H4A	111.4	C9'—C8'—H8'B	110.7
C3—C4—H4B	111.4	C7'—C8'—H8'B	110.7
C5—C4—H4B	111.4	H8'A—C8'—H8'B	108.8
H4A—C4—H4B	109.3	C8'—C9'—C10'	102.2 (9)
N1—C5—C4	104.6 (6)	C8'—C9'—H9'A	111.3
N1—C5—H5A	110.8	C10'—C9'—H9'A	111.3
C4—C5—H5A	110.8	C8'—C9'—H9'B	111.3
N1—C5—H5B	110.8	C10'—C9'—H9'B	111.3
C4—C5—H5B	110.8	H9'A—C9'—H9'B	109.2
H5A—C5—H5B	108.9	N2—C10'—C9'	103.7 (9)
N1—C1'—S2	124.3 (9)	N2—C10'—H10C	111.0
N1—C1'—S1	105.3 (8)	C9'—C10'—H10C	111.0
S2—C1'—S1	128.8 (8)	N2—C10'—H10D	111.0
N1—C2'—C3'	101.0 (8)	C9'—C10'—H10D	111.0
N1—C2'—H2'A	111.6	H10C—C10'—H10D	109.0
C3'—C2'—H2'A	111.6	S3—C11—S1	114.05 (18)
N1—C2'—H2'B	111.6	S3—C11—H11A	108.7
C3'—C2'—H2'B	111.6	S1—C11—H11A	108.7
H2'A—C2'—H2'B	109.4	S3—C11—H11B	108.7
C4'—C3'—C2'	102.0 (9)	S1—C11—H11B	108.7
C4'—C3'—H3'A	111.4	H11A—C11—H11B	107.6
			
C1'—N1—C1—S2	52 (6)	C10'—N2—C6—S4	172.3 (10)
C5'—N1—C1—S2	173.4 (8)	C10—N2—C6—S4	177.1 (8)
C2'—N1—C1—S2	−8.8 (15)	C7—N2—C6—S4	−9.9 (11)
C2—N1—C1—S2	−7.1 (13)	C6'—N2—C6—S3	−70 (4)
C5—N1—C1—S2	179.6 (7)	C7'—N2—C6—S3	−179.6 (10)
C1'—N1—C1—S1	−120 (7)	C10'—N2—C6—S3	4.0 (12)
C5'—N1—C1—S1	0.9 (12)	C10—N2—C6—S3	8.7 (10)
C2'—N1—C1—S1	178.7 (10)	C7—N2—C6—S3	−178.2 (7)
C2—N1—C1—S1	−179.6 (8)	C6'—S4—C6—N2	−114 (4)
C5—N1—C1—S1	7.1 (12)	C6'—S4—C6—S3	54 (3)
C1'—S2—C1—N1	−95 (7)	C6'—S3—C6—N2	106 (4)
C1'—S2—C1—S1	77 (7)	C11—S3—C6—N2	172.9 (4)
C1'—S1—C1—N1	126 (6)	C6'—S3—C6—S4	−63 (4)
C11—S1—C1—N1	173.6 (7)	C11—S3—C6—S4	3.7 (5)
C1'—S1—C1—S2	−47 (6)	C6—N2—C7—C8	−164.2 (6)
C11—S1—C1—S2	1.4 (8)	C6'—N2—C7—C8	−176.1 (7)
C1—N1—C2—C3	−166.2 (8)	C7'—N2—C7—C8	−133 (32)
C1'—N1—C2—C3	−174.8 (9)	C10'—N2—C7—C8	13.9 (13)
C5'—N1—C2—C3	13.3 (13)	C10—N2—C7—C8	9.8 (12)
C2'—N1—C2—C3	−93 (32)	N2—C7—C8—C9	−32.0 (10)
C5—N1—C2—C3	8.1 (11)	C7—C8—C9—C10	42.8 (10)
N1—C2—C3—C4	−29.8 (11)	C6—N2—C10—C9	−170.8 (6)
C2—C3—C4—C5	38.6 (10)	C6'—N2—C10—C9	−158.5 (7)
C1—N1—C5—C4	−169.9 (8)	C7'—N2—C10—C9	17.1 (13)
C1'—N1—C5—C4	−160.6 (9)	C10'—N2—C10—C9	−70 (11)
C5'—N1—C5—C4	−128 (7)	C7—N2—C10—C9	15.6 (11)
C2'—N1—C5—C4	17.5 (11)	C8—C9—C10—N2	−35.2 (10)
C2—N1—C5—C4	15.9 (10)	C6—N2—C6'—S4	−66 (4)
C3—C4—C5—N1	−33.6 (9)	C7'—N2—C6'—S4	10.4 (13)
C1—N1—C1'—S2	−116 (7)	C10'—N2—C6'—S4	−179.0 (9)
C5'—N1—C1'—S2	−179.4 (10)	C10—N2—C6'—S4	−174.4 (8)
C2'—N1—C1'—S2	7.9 (17)	C7—N2—C6'—S4	12.3 (12)
C2—N1—C1'—S2	9.6 (16)	C6—N2—C6'—S3	97 (4)
C5—N1—C1'—S2	−174.2 (8)	C7'—N2—C6'—S3	174.0 (10)
C1—N1—C1'—S1	51 (6)	C10'—N2—C6'—S3	−15.4 (12)
C5'—N1—C1'—S1	−12.6 (13)	C10—N2—C6'—S3	−10.8 (11)
C2'—N1—C1'—S1	174.7 (10)	C7—N2—C6'—S3	175.9 (8)
C2—N1—C1'—S1	176.4 (8)	C6—S4—C6'—N2	49 (3)
C5—N1—C1'—S1	−7.3 (14)	C6—S4—C6'—S3	−111 (4)
C1—S2—C1'—N1	73 (7)	C11—S3—C6'—N2	−174.7 (6)
C1—S2—C1'—S1	−91 (7)	C6—S3—C6'—N2	−60 (4)
C1—S1—C1'—N1	−45 (5)	C11—S3—C6'—S4	−12.9 (7)
C11—S1—C1'—N1	−177.6 (7)	C6—S3—C6'—S4	102 (4)
C1—S1—C1'—S2	121 (7)	C6—N2—C7'—C8'	170.5 (8)
C11—S1—C1'—S2	−11.6 (12)	C6'—N2—C7'—C8'	159.0 (8)
C1—N1—C2'—C3'	166.3 (8)	C10'—N2—C7'—C8'	−12.6 (16)
C1'—N1—C2'—C3'	157.5 (10)	C10—N2—C7'—C8'	−16.7 (15)
C5'—N1—C2'—C3'	−15.8 (15)	C7—N2—C7'—C8'	21 (30)
C2—N1—C2'—C3'	59 (31)	N2—C7'—C8'—C9'	31.1 (14)
C5—N1—C2'—C3'	−20.9 (13)	C7'—C8'—C9'—C10'	−37.1 (14)
N1—C2'—C3'—C4'	35.6 (13)	C6—N2—C10'—C9'	166.5 (7)
C2'—C3'—C4'—C5'	−42.5 (12)	C6'—N2—C10'—C9'	178.5 (7)
C1—N1—C5'—C4'	167.5 (8)	C7'—N2—C10'—C9'	−10.1 (15)
C1'—N1—C5'—C4'	176.6 (9)	C10—N2—C10'—C9'	84 (11)
C2'—N1—C5'—C4'	−10.5 (14)	C7—N2—C10'—C9'	−11.4 (14)
C2—N1—C5'—C4'	−12.0 (12)	C8'—C9'—C10'—N2	28.7 (13)
C5—N1—C5'—C4'	26 (6)	C6'—S3—C11—S1	84.1 (4)
C3'—C4'—C5'—N1	32.6 (11)	C6—S3—C11—S1	76.5 (3)
C6'—N2—C6—S4	98 (4)	C1—S1—C11—S3	79.2 (4)
C7'—N2—C6—S4	−11.3 (12)	C1'—S1—C11—S3	84.7 (5)

### Hydrogen-bond geometry (Å, °)

**Table:**

*D*—H···*A*	*D*—H	H···*A*	*D*···*A*	*D*—H···*A*
C4—H4A···S3^i^	0.99	2.89	3.811 (9)	155
C10—H10B···S4^ii^	0.99	2.99	3.699 (11)	130
C9—H9A···S1^ii^	0.99	2.87	3.740 (8)	147
C9'—H9'A···S1^ii^	0.99	3.50	4.209 (11)	131
C5'—H5'B···S2^i^	0.99	2.94	3.704 (14)	137

Symmetry codes: (i) −*x*+1/2, *y*, *z*+1/2; (ii) −*x*+1, −*y*+1, *z*−1/2.
